# Convolutional autoencoder based model HistoCAE for segmentation of viable tumor regions in liver whole-slide images

**DOI:** 10.1038/s41598-020-80610-9

**Published:** 2021-01-08

**Authors:** Mousumi Roy, Jun Kong, Satyananda Kashyap, Vito Paolo Pastore, Fusheng Wang, Ken C. L. Wong, Vandana Mukherjee

**Affiliations:** 1 Department of Computer Science, Stony Brook University, Stony Brook, NY 11794 USA; 2 Department of Mathematics and Statistics, Georgia State University, Atlanta, GA 30303 USA; 3 IBM Research - Almaden, 650 Harry Rd, San Jose, CA 95120 USA

**Keywords:** Cancer, Cell biology, Computational biology and bioinformatics, Health care, Medical research

## Abstract

Liver cancer is one of the leading causes of cancer deaths in Asia and Africa. It is caused by the Hepatocellular carcinoma (HCC) in almost 90% of all cases. HCC is a malignant tumor and the most common histological type of the primary liver cancers. The detection and evaluation of viable tumor regions in HCC present an important clinical significance since it is a key step to assess response of chemoradiotherapy and tumor cell proportion in genetic tests. Recent advances in computer vision, digital pathology and microscopy imaging enable automatic histopathology image analysis for cancer diagnosis. In this paper, we present a multi-resolution deep learning model HistoCAE for viable tumor segmentation in whole-slide liver histopathology images. We propose convolutional autoencoder (CAE) based framework with a customized reconstruction loss function for image reconstruction, followed by a classification module to classify each image patch as tumor versus non-tumor. The resulting patch-based prediction results are spatially combined to generate the final segmentation result for each WSI. Additionally, the spatially organized encoded feature map derived from small image patches is used to compress the gigapixel whole-slide images. Our proposed model presents superior performance to other benchmark models with extensive experiments, suggesting its efficacy for viable tumor area segmentation with liver whole-slide images.

## Introduction

Liver is a visceral organ frequently targeted by cancer metastasis. Hepatocellular carcinoma (HCC) is the most common histological type of primary liver cancers with hepatocellular differentiation. Tumors are known to have multiple cellular and stromal components such as, tumor cells, inflammatory cells, blood vessels, acellular matrix, tumor capsule, fluid, mucin, or necrosis. The viable tumor regions are more active and responsive regions inside the tissue area. In clinical practice, tissue samples are used to assess chemoradiotherapy response rates and tumor cell proportions in genetic tests. Therefore, there is a strong but unmet need to accurately evaluate viable tumor. Pathologists often use a semi-quantitative grading system for the residual tumor burden estimation. Thanks to recent advancement in digital pathology, whole slide images (WSI) of tumor tissues can now be quantitatively and automatically analyzed with microscopy image analysis algorithms that complement traditional manual tissue examinations^1^. MICCAI grand challenge 2019 has prepared a well-annotated dataset to address this specific problem^2^. With the recent emergence of deep learning methods for medical image analysis, tumor segmentation in liver WSIs can be well addressed by deep learning models that conduct patch-wise classifications or pixel-wise semantic segmentation. The most popular semantic segmentation framework is FCN^3^ consisting of down-sampling layers to extract image features and up-sampling layers to generate the segmentation mask. UNet^4^ is another widely used segmentation model introducing the skip connections from down-sampling layers to up-sampling layers to preserve the information for high-resolution images. By contrast, the patch-based methods partition the large WSIs into small image patches and classify each patch as either tumor or non-tumor^5,6^. A CNN is used^7^ to extract features from each patch and assign a prediction score. Based on the prediction score map in WSIs, breast metastasis cancer is segmented. As contextual information from one fixed resolution may not be enough to detect the tumor regions accurately, multiple methods have addressed this shortcoming by incorporating multi-scale contextual information into the patch-wise classification model^8^. Convolutional auto-encoder (CAE) and Convolutional neural network (CNN) have been integrated for finger vein verification, where CAE is used to learn the feature codes from finger vein images and the CNN is used to classify finger vein from the learned feature codes^9^. A multi-model network G-EyeNet is proposed with a deep CAE and a traditional CNN^10^. These two components jointly optimize the network to minimize both image reconstruction and classification error based on a multi-task learning procedure. An unsupervised method is proposed to find contrast patterns between normal and malignant images^11^. However, these existing methods are not sufficient for viable tumor segmentation, as they use the standard CAE structure designed with standard MSE based loss function. We notice MSE loss by itself results in blurry reconstructed images that lead to a lower patch-based tumor classification accuracy. In addition, these frameworks have mostly single resolution and are implemented on low resolution images. For our histopathology dataset, we need to incorporate more spatial details as well as detailed structural information. Therefore, multi-resolution based framework is preferred.

In this study, we propose an automated deep learning-based model, named HistoCAE, for segmentation of viable tumors in liver WSIs. Specifically, we establish a multi-resolution CAE based framework for image reconstruction, followed by a classification module that labels each image patch as either tumor or non-tumor. The resulting patch-based prediction results are assembled spatially to generate the final segmented tumor regions in a WSI. Unlike these aforementioned methods, we propose a customized reconstruction loss function for better image reconstruction and tumor segmentation. Besides, our architecture can incorporate features from multi-resolution images for tumor segmentation. We systematically compare the segmentation performance of our model with that of benchmark models (i.e. ResNet-101 and U-Net) at multiple image magnifications. Our proposed model presents superior performance than other benchmark models, suggesting its efficacy for viable tumor segmentation using liver WSIs.

## Methods

### Architecture of HistoCAE

**Figure 1:**
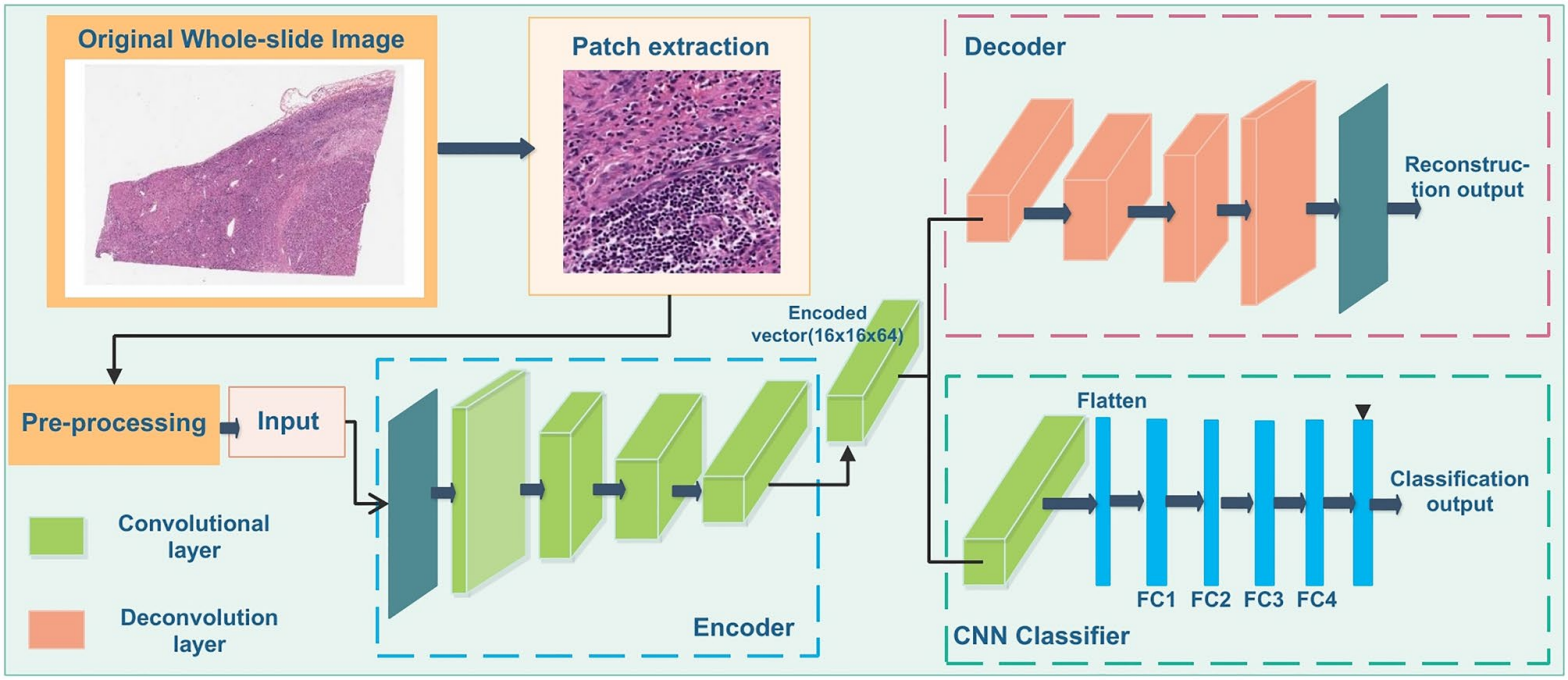
Schema of model architecture: HistoCAE model consists of an Encoder-Deocder module for patch reconstruction and a CNN Classifier module for tumor classification at 10 × image magnification.

We combine supervised and unsupervised methods for learning class conditional data-driven feature distributions and propose a convolutional autoencoder (CAE) based method to learn the structural feature of liver histopathology images. Our proposed HistoCAE network consists of an autoencoder module ${h}_{CAE}=\left\{{h}_{E}, {h}_{D}\right\}$ for learning image features and a classifier module ${h}_{CL}=\left\{{h}_{E}, { h}_{C}\right\}$ for supervised classification. The encoder component ${h}_{E}$ has four convolutional layers, while the decoder ${h}_{D}$ has four deconvolution layers. Each convolutional layer consists of a convolution operation (stride 1) with ReLU as non-linear activation function followed by batch normalization and a second convolution operation (stride 2) with ReLU activation followed by batch normalization. Instead of using pooling operation, we explicitly use stride 2 to reduce the feature map size after each convolutional layer. In our study, we notice that pooling operations cause spatial information loss, leading to deteriorated image reconstruction results. The decoder module ${h}_{D}$ is trained to reconstruct the original input with the encoded latent vector from the encoder ${h}_{E}$. The filter used for convolution operation is of $3\times 3$ in size. The depth of each layer in the encoder module ${h}_{E}$ is 16, 32, 64 and 64, respectively. The last layer represents the bottleneck layer. The encoded latent vector at the bottleneck block has the dimension of $16 \times 16 \times 64$. The bottleneck block is connected to the classifier module ${h}_{C}$ that has one convolutional layer. A softmax classifier is associated with the classifier module ${h}_{C}$ that classifies each encoded vector from ${h}_{E}$ in the latent space. The HistoCAE network structure is illustrated in Fig. 1. The detailed description of the network structure is presented in Table S1 (Supplement).

### Reconstruction loss formulation

For image reconstruction, the loss function plays a significant role for the reconstruction result quality assessment. The reconstruction loss ${l}_{R}$ is usually defined by the mean squared error (MSE) that measures the differences between the input and reconstructed image:

1 $$ l_{MSE} = \mathop \sum \limits_{i = 1}^{M} \mathop \sum \limits_{j = 1}^{N} \left( {x_{ij} - \hat{x}_{ij} } \right)^{2} $$

where $M$ is the total number of images in the batch, $N$ is the number of pixels in each input image, ${x}_{ij}$ represents the $j$-th pixel value of $i$-th original image and ${\widehat{x}}_{ij}$ represents the value of $j$-th pixel of $i$-th reconstructed image. However, MSE loss causes the generation of a blurry reconstructed image with significant structural information loss from the original image^12^. As the cellular structures provides key information to liver cancer diagnosis^11^, we make autoencoders learn and retain such key information by including the Structural Similarity (SSIM) index in the loss function: ${l}_{SSIM}\left({x}_{ij}, {\widehat{x}}_{ij}\right)=1-SSIM({x}_{ij}, {\widehat{x}}_{ij})$. The SSIM based loss term encourages autoencoders to maintain the structural information in image patches:

2 $$SSIM\left(x, \widehat{x}\right)=\left[l{\left(x, \widehat{x}\right)}^{\alpha } \cdot c{\left(x, \widehat{x}\right)}^{\beta } \cdot s{\left(x, \widehat{x}\right)}^{\gamma }\right] $$

where $l(\cdot )$, $c(\cdot )$ and $s(\cdot )$ in Eq. 2 are the luminance, contrast and structural comparison functions, respectively; $\alpha , \beta$ and $\gamma$ represent the component weights ^11^.

We compare the performance of models trained with different combinations of loss functions and experimentally find the optimal joint reconstruction loss function ${l}_{R}$ defined as a a weighted sum of MSE, SSIM and Mean Absolute Error (MAE): ${l}_{R}= \alpha \times {l}_{MSE} + \beta \times {l}_{SSIM} + \gamma \times {l}_{MAE}$ where, the weight values $\alpha , \beta$ and $\gamma$ are hyper-parameters determined by rigorous experimentations as $\alpha$ = $\beta$ = $\gamma$  = 0.5. Here, $l_{MAE}$ is defined as

3 $$ l_{MAE} = { }\frac{1}{M}{ }\frac{1}{N}{ }\mathop \sum \limits_{i = 1}^{M} \mathop \sum \limits_{j = 1}^{N} \left| {x_{ij} { } - { }\hat{x}_{{ij{ }}} } \right| $$

As regular CNN architectures flatten a two-dimensional image to a vector representation, they are subject to spatial information loss and do not fully learn the image feature representations. Similarly, a typical vanilla autoencoder itself is subject to spatial information loss, thus not suitable for complex image feature learning and image reconstruction due to its shallow structure. By contrast, a CAE is equipped with a sliding window and multiple hidden layers in the network essential for learning the spatial features accurately. Additionally, a CAE is good at learning image feature representations in a much lower dimensional feature space via several sequential layers of convolutional operations and striding. In our study, the original patch size of 256 × 256 × 3 is reduced to 16 × 16 × 64 after the encoding computation. The resulting feature maps from patches can be organized by the original spatial arrangement as depicted in Fig. S1(A) (Supplement). In this way, it generates the compressed representation (i.e. the feature map) of a gigapixel whole-slide image and retains the semantic information in a lower dimensional space^13^. As a way to assess the CAE learning performance, the image reconstruction performances of models trained with different reconstruction loss functions are measured by SSIM and presented in Fig. S1(B) (Supplement).

### Giga-pixel image compression by encoded feature map representation

Another benefit of using autoencoder based classifier HistoCAE is to generate the compressed representation of gigapixel whole-slide images. A giga-pixel image is a 3-dimensional array typically with more than one billion pixels. Such images are widely captured and used in the field of computational pathology, remote sensing, satellite imaging among others. It is computationally infeasible to feed the whole slide image data to a CNN model directly for training. Therefore, the encoder-decoder module ${h}_{CAE}$ of HistoCAE can help compress a giga-pixel image into a highly compact representation for further analysis. This size reduction is achieved by mapping images from low-level pixel space to a higher-level latent space using neural networks^13^. The goal is to retain the semantic information with a reduced representation of a giga-pixel image by shrinking its spatial dimensions and growing along the feature directions as depicted in Fig. S1(A) (Supplement). This method works as follows. First, a gigapixel image is divided into a set of high-resolution patches. Next, each high-resolution patch is compressed using a neural network (e.g. ${h}_{E}$ in our model) that maps every image into a lower-dimensional embedding feature map. Finally, each feature map can be placed into an array according to the original spatial arrangement such that the neighboring feature map in the array represents the neighbor patches in the original image. CAE is one type of autoencoder specifically designed to learn the compact representation of a given image data manifold. In our study, we use the encoded feature map ($16\times 16\times 64$ in size) generated from ${h}_{E}$ of ${h}_{CAE}$ module to represent the corresponding high-resolution image of $256\times 256\times 3$ in size. The lower-dimensional feature vectors generated from ${h}_{E}$ module can remove the spurious details from patches. By this technique, the gigapixel images can be reduced by size before they are provided to a CNN model for further analysis by transfer learning. We represent a giga-pixel whole-slide image as $I \in {\mathbb{R}}^{M\times N\times 3}$ where $M$ and $N$ represent the total number of rows and columns in $I$. We compress $I$ into a more compact representation $I\mathrm{^{\prime}}$ by two steps. First, the WSI $I$ is divided into set of several high-resolution patches $P=\{{p}_{ij}\}$ where ${p}_{ij}$ is sampled from the $i$ th row and $j$ th column of a uniform grid of square patches of size $256$ with a stride value of $256$ throughout $I$. Next, each patch ${p}_{ij}$ is compressed as a set of low-dimensional encoded feature maps of size $16\times 16\times 64$ independently by the ${h}_{CAE}$ module. This is a more powerful technique for image compression compared to the classical approaches^14^. Although some studies^15,16^ have provided to other networks the extracted features from neural network in the context of transfer learning and representation learning, we exploit the extracted features from the encoder-decoder module ${h}_{CAE}$ to perform viable tumor segmentation task in this study.

### Viable tumor segmentation

The extracted feature map from the encoder is provided to the CNN network ${h}_{C}$ that is followed by a softmax classifier for final classification. We use $0.7$ dropout value for the fully connected layers in the CNN module to prevent overfitting. This technique can also help reduce the training parameters by randomly omitting hidden neurons. The probability of random neuron omission is another hyper-parameter to be set manually. We set it to be $0.7$ in this study. The classification loss is a cross-entropy function that measures the difference between the actual and prediction label of the image: ${l}_{C}= - \frac{1}{M} \sum_{c=1}^{M}\left({y}_{c}\mathrm{log}\left({\widehat{y}}_{c}\right)\right)$ where $M$ is the total number of images in the current batch; ${y}_{c}$ and ${\widehat{y}}_{c}$ represent the actual and predicted label of the $c$-th image in the batch. The learned weights for encoder ${h}_{E}$ during encoder-decoder module ${h}_{CAE}$ training are used to initialize the weights of encoder ${h}_{E}$ for encoder-classifier module ${h}_{CL}$ training and next update the whole ${h}_{CL}$ module without fixing the encoder ${h}_{E}$ weights. Finally, the patch-based prediction results from HistoCAE model are spatially combined to generate the tumor segmentation masks for viable tumor regions in a WSI.

### Multi-resolution extension: MR-HistoCAE

As a step further, we next aggregate contextual information from patches of 5 ×, 10 × and 20 × magnifications to generate tumor segmentation result at the highest magnification (i.e. 20 ×) that retains finer tissue histology details on tumor edges. This Multi-Resolution HistoCAE network (MR-HistoCAE) helps leverage contexts from both wide field-of-views and high-magnification images with tissue details useful for accurate viable tumor region detection.

**Figure 2:**
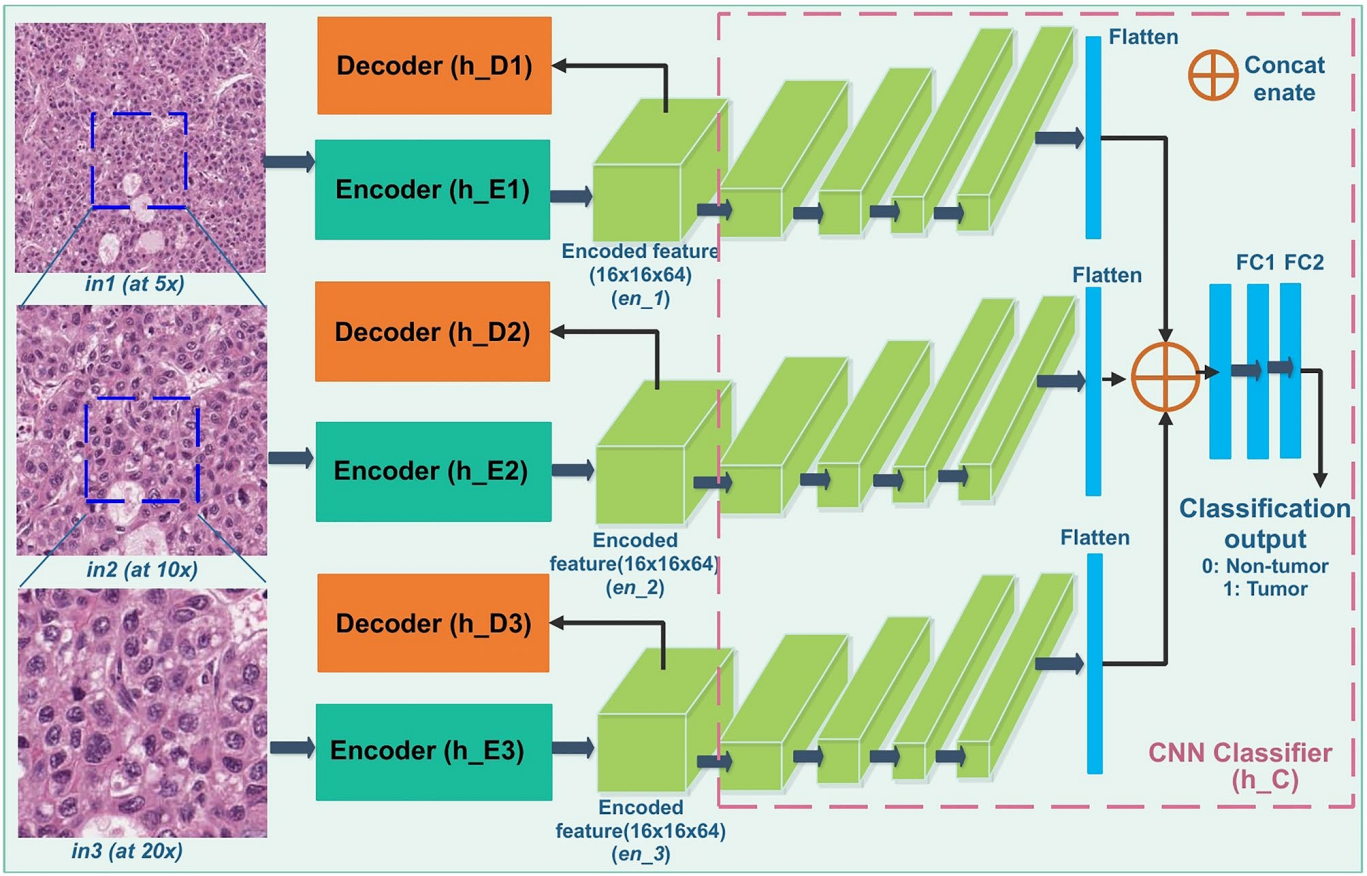
MRHistoCAE model aggregates patches at 5 × , 10 × and 20 × , respectively. The CAE module is same as the HistoCAE, but with the modified Classifier.

In this model, three different encoder-decoder modules ${h}_{CAE}^{1}$, ${h}_{CAE}^{2}$ and ${h}_{CAE}^{3}$ are used to reconstruct the original image patches at 5 ×, 10 × and 20 × magnifications, respectively. Image patches centered at the same location are extracted from three different magnifications. The resulting ground truth tumor masks are produced for three magnifications. Encoded feature maps from the bottleneck block of the separately trained autoencoders are passed to the classifier module ${h}_{C}$. Specifically, each encoded feature map is connected to four convolutional layers. Each convolutional layer consists of one convolution operation with ReLU activation and stride 2, and the batch normalization. The sizes of the filters in the four convolutional layers are 128, 256, 512 and 512, respectively. The output of last convolution layer is flattened into a vector of $512$ in length. All three flattened vectors are concatenated into a single vector of length $1536$ that is further connected to three fully connected dense layers. Each dense layer consists of a ReLU activation, a batch normalization, and a dropout layer. The last fully connected layer has a softmax function used for class label prediction. The dropout layer is applied only during the training stage and turned off for testing. Figure 2 demonstrates the schema of the proposed MR-HistoCAE model architecture. The MR-HistoCAE model is trained for multiple epochs to minimize both reconstruction and classification loss by the multi-task learning method^17^. Patch-based prediction results are finally assembled to generate the tumor segmentation mask for each WSI at the highest magnification (i.e. 20 ×).

## Results

### Datasets and evaluation metrics

The dataset for this work comes from PAIP challenge 2019 that is a part of MICCAI 2019 grand challenges^2^. It includes 50 fully annotated H&E WSIs of liver tissue at 20 × magnification. Fully annotated viable tumor area are provided by expert pathologists from Seoul National University Hospital, South Korea. All WSIs are stained by H&E and scanned by Aperio AT2 at × 20 power. Specifically, we follow the general guideline and split these images roughly into 70:20:10 for training, validation, and testing. Thus, we use 36 WSIs for training, eight for validation, and remaining six for testing. Due to their giga-pixel scale, WSIs cannot be directly provided to the deep learning models. As a pre-processing step, each WSI is partitioned into non-overlapping patches of size $256\times 256$ at 5 ×, 10 × and 20 ×, respectively. Since we design the segmentation task as a patch-wise classification, we need to generate the ground truth label from the viable tumor mask. A patch is only labeled as *tumor* if 30% or more of its region is covered by the tumor pixels. Otherwise, it is labeled as *non-tumor*. This is also considered as a pre-processing step. Dividing the image into patches also helps models learn local tumor environment, such as tumor cells, inflammatory cells, neoplastic vasculatures, and other small-scale histology structures. In total, 60,000 patches are used for training, 12,000 for validation and 21,019 for testing. No patches from training image data is used for validation or testing. We apply our tumor segmentation pipeline to images at each magnification separately. Additionally, we extend our framework to incorporate all three different magnification and develop multi-resolution framework for generating the segmentation map at the highest magnification, i.e. 20 ×.

All pre-processing steps are performed with MATLAB 2018b software package^18^. After the deep learning-based model training, the spatially ordered patch-based prediction results are assembled to generate the segmentation result for WSIs. This step is illustrated in Fig. S4 (Supplement). All the data processing pipelines, and model training modules are run on IBM cluster with multiple CPU cores and 2 NVIDIA Tesla V100 GPUs. HistoCAE and baseline models, e.g. ResNet-101 and U-Net, are implemented on Keras and Tensorflow backend^19^ with Tensorflow Version 1.9.0 and Tensorflow-GPU Version 1.13.1. For the encoder-decoder module training, data augmentation is implemented with Keras standard data augmentation library for random rotation, horizontal and vertical flipping. The decoder and the softmax classifier are configured by ADAM^20^ optimizer with a learning rate of 0.0001 and beta value of 0.05. Five-fold cross-validation method is used during the training process. The encoder-decoder module ${h}_{CAE}$ is trained for 200 epochs with batch size of 64. This is followed by the encoder-classifier module ${h}_{CL}$ training for another 200 epochs with the same batch size. The classification results are evaluated with four metrics, namely precision, recall, F1-score, and testing accuracy. Testing accuracy is the ratio of number of correctly classified patches to the total number of patches in the test set. The WSI viable tumor segmentation performance is evaluated by the overall dice similarity score. The overall workflow is visually presented in Fig. S4 (Supplement).

### Experimental results: quantitative comparison

We present in Table 1 the patch-wise classification as well as WSI tumor segmentation performance of HistoCAE, its variations, benchmark models for classification (i.e. ResNet-101^21^, VGGNet^22^, Google’s Inception V3^23^, and DenseNet^24^) and state-of-the-art segmentation models (i.e. UNet^4^, SegNet^25^, DeepLabV3^26^, PSPNet^27^, RefineNet^28^ and MobileUNet^29^) at 10 × magnification. The overall performances of segmentation models are worse than those of the classification models, due in large part to the complex vasculature structures in tumor masks. It is also noticed that tumor masks have irregular shapes, making it difficult to train the end-to-end segmentation pipeline. We used morphological operation to close the small blob regions less than or equal to 50 pixels in the ground truth mask before training the segmentation models. We carefully find this cutoff value so that we do not lose tumor structures or small vascular regions. As viable tumor regions are large in size, scattered over tissue domains, and do not show obvious patterns, we choose to construct the viable tumor segmentation mask with the patch-wise classification results. In HistoCAE model, the autoencoder helps initialize the classifier module ${h}_{CL}$ with proper weights through the prior unsupervised training of ${h}_{CAE}$ module. Performances of classification model Inception V3 and DenseNet are comparable to that of our model HistoCAE, although the final viable tumor segmentation performance of HistoCAE1 is better than DenseNet and Inception V3 by the dice similarity coefficient. However, DenseNet and Inception V3 have more complex and deeper network structures and need a much larger set of training data (e.g. ImageNet dataset etc.) without overfitting due to the large number of trainable parameters. In practice, it is often necessary to use pre-trained weights to initialize such deep models and proceed with fine tuning for specific datasets especially when used for tasks with small sized dataset such as ours. By contrast, our model produces comparable performance, but is much more simplified with substantially fewer number of network parameters than the state-of-the-art models. Therefore, our model is more robust for tumor segmentation in WSIs than other state-of-the-art methods. This is confirmed by a higher dice similarity value by experiments. Thus, our model is promising to be readily extended to other domain data without pre-training.

**Table 1:** Quantitative evaluation of patch-based classification and tumor segmentation using 10 × images.

	Model name	Test accuracy	Precision		Recall		F1-Score		Proportion of correct patches	Dice similarity
			Non-tumor	Tumor	Non-tumor	Tumor	Non-tumor	Tumor		
Variations of our model	HistoCAE1 (MSE + SSIM + MAE loss)	0.95	0.96	0.93	0.95	0.95	0.96	0.94	0.94	0.87
	HistoCAE2 (MSE loss)	0.93	0.95	0.91	0.93	0.93	0.94	0.92	0.93	0.83
	HistoCAE3 (SSIM loss)	0.93	0.95	0.91	0.93	0.94	0.94	0.92	0.93	0.56
	HistoCAE4 (MAE loss)	0.91	0.94	0.88	0.91	0.92	0.92	0.90	0.91	0.72
	HistoCAE5 (MSE + SSIM loss)	0.92	0.94	0.91	0.93	0.91	0.93	0.91	0.92	0.72
	HistoCAE6 (MSE + MAE loss)	0.93	0.94	0.94	0.96	0.91	0.95	0.93	0.93	0.77
	HistoCAE7 (SSIM + MAE loss)	0.93	0.94	0.93	0.95	0.92	0.95	0.93	0.93	0.76
Classification models	ResNet-101	0.93	0.96	0.91	0.93	0.94	0.95	0.93	0.94	0.84
	Vgg19	0.57	0.57	0	1	0	0.73	0	–	–
	DenseNet	0.96	0.95	0.94	0.97	0.94	0.96	0.95	–	0.80
	InceptionV3	0.95	0.95	0.96	0.97	0.93	0.96	0.95	–	0.77
Segmentation models	U-Net	–	–	–	–	–	–	–	–	0.59
	SegNet	0.82	0.80		0.82		0.77		–	0.73
	DeepLabV3	0.73	0.88		0.73		0.72		–	0.67
	PSPNet	0.79	0.88		0.79		0.78		–	0.72
	RefineNet	0.84	0.89		0.84		0.83		–	0.75
	Mobile-UNet	0.86	0.93		0.86		0.86		–	0.77

Note that models HistoCAE1, HistoCAE2, HistoCAE3, HistoCAE4, HistoCAE5, HistoCAE6 and HistoCAE7 are trained with reconstruction loss functions ${l}_{R}$ (i.e. MSE + SSIM + MAE), ${l}_{MSE}$, ${l}_{SSIM}$, ${l}_{MAE}$, ${l}_{MSE+SSIM}$, ${l}_{MSE+MAE}$, ${l}_{SSIM+MAE}$ respectively. HistoCAE1 presents the best overall performance, due to the design of the balanced loss function. Such a loss function involving multiple loss components enables an enhanced feature distribution learning. Additionally, inclusion of SSIM loss in the reconstruction loss facilitates improved spatial information learning. When we only use either MSE, SSIM, or MAE loss alone, the resulting classification and tumor segmentation performance becomes worse. When we gradually add the terms, the resulting performance is improved. For instance, the loss function of MSE + SSIM produces accuracy of 0.92 whereas the loss function of MSE + SSIM + MAE increases the accuracy to 0.95. Similarly, the WSI tumor segmentation performance assessed by dice value is improved from 0.56 for SSIM loss to 0.76 for SSIM + MAE based loss, and finally to 0.87 for MSE + SSIM + MAE based loss.

With the HistoCAE model, we compare the classification and viable tumor segmentation performance at 5 ×, 10 × and 20 × magnifications, and notice that the best patch-based classification and WSI tumor segmentation accuracy is achieved at 10 × magnification. The quantitative comparison result is shown in Table S2 (Supplement). The model at 10 × resolution achieves the best performance by precision, recall, F1-score and dice similarity consistently. Therefore, we chose 10 × resolution for HistoCAE model. For the same reason, we compare HistoCAE model and all variations with state-of-the art methods for classification and segmentation performance by evaluation metrics at 10 × magnification. We further spatially assemble the patch-based prediction results to generate tumor segmentation masks at 10 × magnification. Tumor boundaries are found to be coarse.

Thus, we test our multi-resolution model MR-HistoCAE at the highest magnification (i.e. 20 ×) that retains finer tissue histology details on tissue edges. The quantitative comparison result is presented in Table S3 (Supplement). Supplementary Table S3 suggests that MR-HistoCAE has better performance than the HistoCAE at 20 × . It also suggests that, MR-HistoCAE has comparable performance to HistoCAE at 10 × by all evaluation metrics. The resulting F1-scores from the MR-HistoCAE model with reconstruction loss ${l}_{MSE}$ are 0.95 and 0.91 for non-tumor and tumor regions at 20 ×, respectively. The overall MR-HistoCAE classification accuracy at 20 × is 0.94. By contrast, HistoCAE1 only yields 0.89 overall classification accuracy at 20 ×. By the quantitative results shown in Table S3 (Supplement), the most significant benefit of using MR-HistoCAE is that it can identify the finer boundaries at a much higher resolution (20 × versus 10 × ) while maintaining the same accuracy as the best performing HistoCAE.

### Experimental results: visual comparison

**Figure 3:**
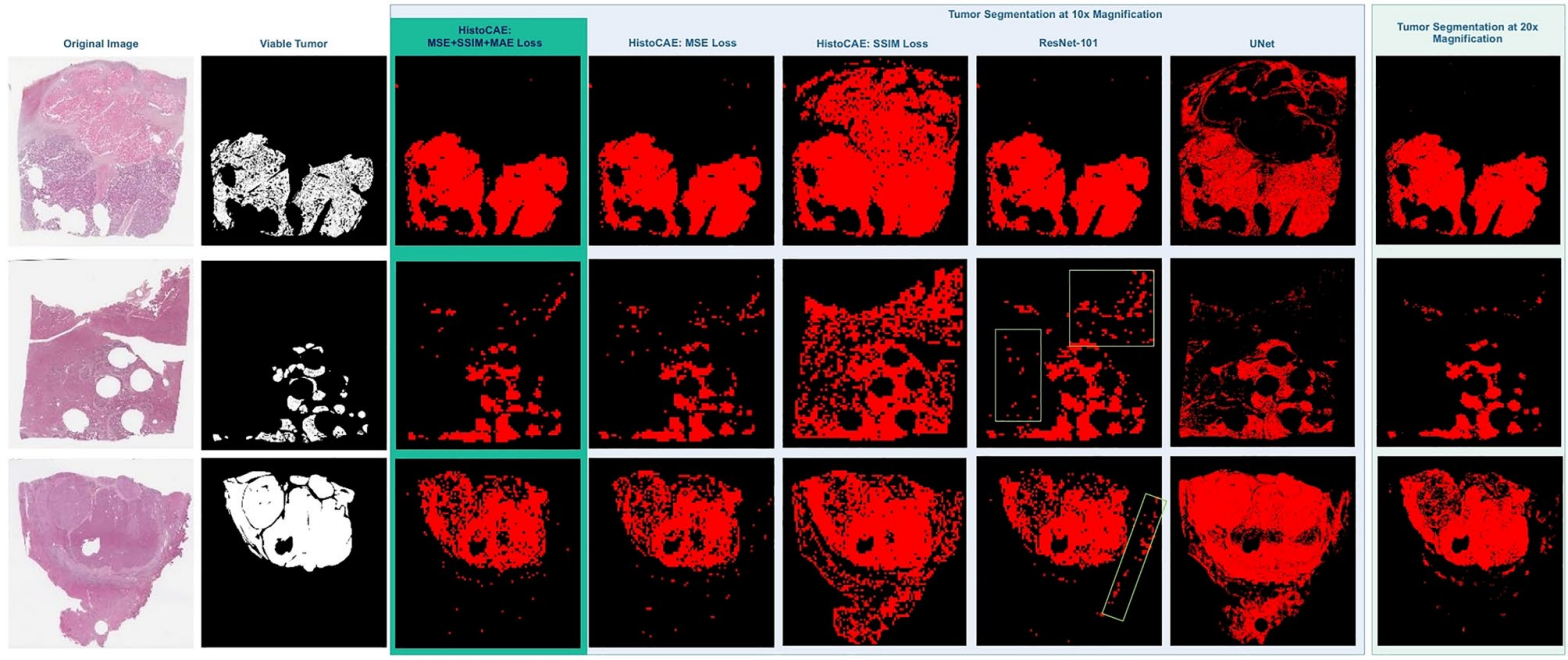
Result comparison with proposed method and benchmark models: (**a**) Original WSIs; (**b**) Ground truth tumor mask; 10 × Segmentation result from (**c**) HistoCAE1, (**d**) HistoCAE2, (**e**) HistoCAE3, (**f**) ResNet-101 (false positive in green boxes), and (**g**) U-Net; (**h**) Segmentation result of MR-HistoCAE at 20 × magnification.

Figure 3 presents the visual comparison results from HistoCAE1, HistoCAE2, HistoCAE3, and baseline models for tumor segmentation in liver tissue WSIs. The classified patches are combined spatially to generate the whole slide tumor segmentation result for each model. Both tissue samples with connected tumor regions and those with numerous small scattered tumor regions are included in Fig. 3. We conclude from the visual results and low Dice Similarity in Table 1 that HistoCAE2, HistoCAE3 and the benchmark models tend to fail to match the ground truth mask. By contrast, the best model HistoCAE1 is able to produce results similar to the ground truth image and preserves tumor region shapes. The false positive regions are shown by green boxes in Fig. 3.

**Figure 4:**
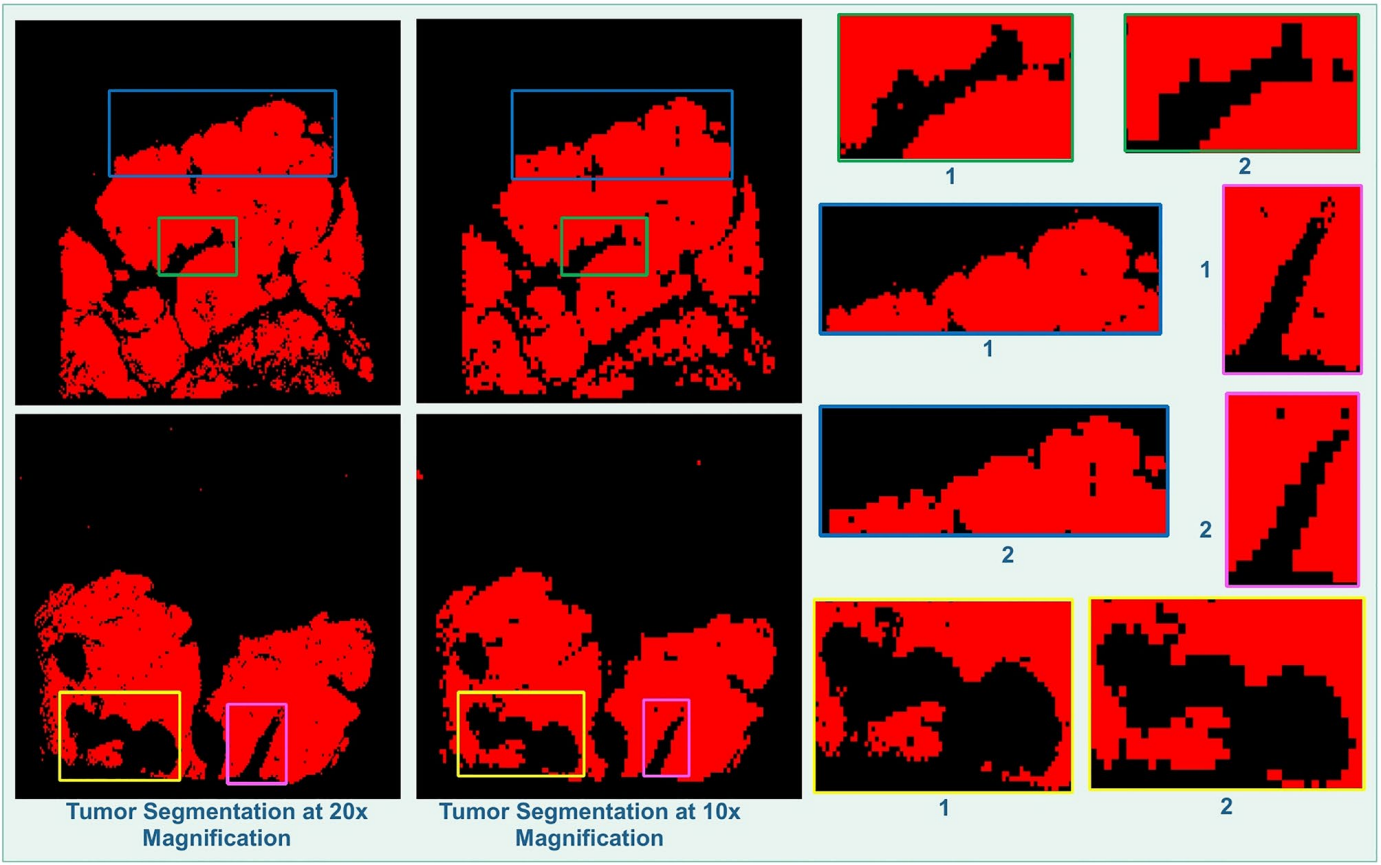
Identified tumor borders (left) by MR-HistoCAE at 20 × ; (middle) by HistoCAE1 at 10 × ; (right) in close-up views for comparison.

In Fig. 4, we demonstrate tumor borders identified at 20 × magnification by MR-HistoCAE model with contextual and structural information from image patches at multiple magnifications (left) and results from HistoCAE1 at 10 × magnification (middle). We notice that MR-HistoCAE is able to capture finer details of tumor edges in Fig. 4. (right) and retain more correctly classified regions at 20 × than 10 × magnification. The predicted tumor regions at 20 × by multi-resolution based patch combination strategy are more similar to the ground truth than that from HistoCAE1, as HistoCAE1 is limited to information from a single 10 × magnification.

## Discussion

We propose a Convolutional Autoencoder (CAE) based classification model HistoCAE for segmentation of viable tumor in liver WSIs. The autoencoder module is trained for image reconstruction. The resulting feature map from each image patch through learning is used for tumor/non-tumor classification. The patch-based prediction result is spatially combined to generate the final tumor segmentation output for each WSI. To optimize CAE learning performance, we design a reconstruction loss function as a weighted sum of MSE, SSIM and MAE loss. This customized loss function helps better reconstruct the images by retaining detailed structural information. By contrast, reconstructed images from standard MSE based loss are blurred.

We further extend our model from HistoCAE to MR-HistoCAE by leveraging multi-resolution tissue information. With multi-field views, it helps incorporate more spatial and detailed cellular information, resulting in substantially finer tumor borders recovery. Our models trained with different loss functions are intensively compared with state-of-the-art classification models, i.e. ResNet-101, VGGNet, Google’s Inception V3, DenseNet and state-of-the-art segmentation models such as, U-Net, SegNet, DeepLabV3, PSPNet, RefineNet and MobileUNet. The superiority of our model performance suggests it is promising for accurate segmentation of liver tumors in histopathology WSIs clinically essential to personalized liver tumor treatment.

In this study, we present how supervised learning task (e.g. patch-based classification) can be achieved by leveraging unsupervised learning methods (e.g. CAE) and how the whole-slide image segmentation can be aggregated from patch-based classification results. To our best knowledge, the application of unsupervised learning models (e.g. CAE) for segmentation of liver tumors in WSIs is not addressed in previous work. We are among the first to show that the application of autoencoder based classifier can be used for patch-based classification in a viable tumor segmentation task with whole-slide histopathology images. Unlike the standard autoencoder structures^30^, our customized ${h}_{CAE}$ module uses convolutional layers with stride 2 to reduce the feature map size. Compared with pooling operation resulting in information loss in each down-sampling stage, our method helps retain spatial information more accurately. We notice that pooling operations cause spatial information loss, leading to deteriorated image reconstruction results. Fig. S3 (Supplement) demonstrates that max-pooling based reconstruction is not able to recover any patch information in some cases and sometimes there is a rippling effect in the reconstructed image.

The poor reconstruction result, in turn, decreases the classification accuracy. The convolution layer can proactively and automatically learn properties, while pooling is a cheaper operation than convolution, both in terms of computation time and the number of parameters to be optimized. Max pooling (or other pooling operations) is a fixed operation. Replacing it with a strided convolution helps enhance the model’s expressiveness ability. The quantitative comparison results between max-pooling vs strided convolution are shown in Table S4(Supplement).

The segmentation of viable tumor regions in WSI using the proposed patch-based classification strategy is leveraged instead of direct segmentation as the state-of-the-art segmentation models are found inferior to patch-based classification strategy for our data. We conducted direct segmentation with several state-of-the-art segmentation frameworks, such as UNet, SegNet, DeepLabV3, RefineNet, PSPNet and MobileUnet. The quantitative performance results are shown in the Table 1. We observe severe performance drops associated with direct segmentation methods when they are compared to the classification-based models. Very often, viable tumors are large in size, have irregular shapes, and are found with a large number of internal discontinuous spaces, making it difficult to train the end-to-end segmentation pipeline. By the quantitative results, we notice that highly irregular vasculatures in the tumor masks devastate performances of methods using the pixel-wise segmentation strategy, such as U-Net. The resulting pixel-wise segmentation prediction accuracy by these methods is substantially contaminated by the presence of a large number of small tissue gaps and small vascular components (black regions) scattered over the viable tumor regions (red mask) as illustrated in Fig. S2 (Supplement). By the dice score, all segmentation methods we compared with have worse performance than the patch-based classification strategy. This well justifies our patch-based classification approach for segmentation of such irregular and disconnected tumor regions.

We compared the performance variance of state-of-the-art classification and segmentation models and HistoCAE by ANOVA and Kruskal–Wallis method. Performing one-way ANOVA and Kruskal–Wallis for each metric across all models, we observe from Table S5 (Supplement) *p*-value < 0.05 for all metrics but dice similarity for ANOVA. When we perform pair-wise comparison, we observe there is significant difference between the segmentation (like DeepLab, PSPNet etc.) and classification models (DenseNet, ResnNet, HistoCAE1). However, the classification models do not show significant difference among themselves. Therefore, there is no statistically significant difference between HistoCAE1 (our model) and any of the classification models (DenseNet, Inception V3 etc.). However, the result difference is statistically significant when we compare HistoCAE1 with segmentation models (DeepLab, PSPNet etc.). For direct visual comparisons, the box plots of different metrics are demonstrated in Fig. S5 (Supplement).

We compare the performance of HistoCAE with its variations. We report the reconstruction accuracy of ${h}_{CAE}$ module by computing the SSIM value for each pair of images. The reconstructed images associated with three different loss functions ${l}_{R}$, ${l}_{MSE}$ and ${l}_{SSIM}$ are illustrated in Fig. S1(B) (Supplement). The high SSIM value (SSIM value 0.78 for one such example patch) by ${l}_{SSIM}$ ensures the benefit of storing the low dimensional embedding vector that can save storage space. Large WSIs can thus be compressed and reconstructed from the encoded map. In future work, we will extend our patch-based classification approach further by replacing autoencoder structure with the generative adversarial network. The discriminator of the generative adversarial network can be designed to classify each patch as real or fake and tumor vs non-tumor at the same time whereas the goal of the generator module is to reconstruct the image patch as accurately as possible to confuse the discriminator. We will also dive deep into more complex patch combining strategy for multi-resolution patch-based result combination. Furthermore, we will investigate the efficacy of our HistoCAE model with other biomedical imaging data.

## Supplementary Information

Supplementary Information.

## Data Availability

The dataset is available at public repository of PAIP 2019 challenge website. The code and implementation details are available in github^31^.
