# Supplementary material for: Convolutional autoencoder based model HistoCAE for segmentation of viable tumor regions in liver whole-slide images
Source: Sci Rep. 2021 Jan 8;11:139. doi: 10.1038/s41598-020-80610-9 (PMC7794421; doi:10.1038/s41598-020-80610-9)
Supplement: Supplementary file 1 — Supplementary Information. [file 41598_2020_80610_MOESM1_ESM.pdf]

Supplementary Information

Convolutional Autoencoder Based Model HistoCAE for Segmentation of Viable Tumor Regions in Liver Whole-Slide Images

Mousumi Roy, Jun Kong, Satyananda Kashyap, Vito Paolo Pastore, Fusheng Wang, Ken C. L. Wong and Vandana Mukherjee

In Fig. 3, we present (a) the original images, (b) reconstructed image from strided convolution, and (c) reconstructed image from max pooling in the encoder module, respectively. The reconstructed image quality in (b) is much better than (c). We also notice that it is not able to recover any patch information by max pooling in some cases. Additionally, there is some rippling effect in the reconstructed image by max pooling.

We quantitatively compare the performance of our method with strided convolution and max pooling in Table 4 . The classification accuracy for strided convolution is consistently higher than that for max pooling by all metrics. The much higher value of dice similarity coefficient also indicates that our method can better segment tumor region than the one with max pooling based encoder.

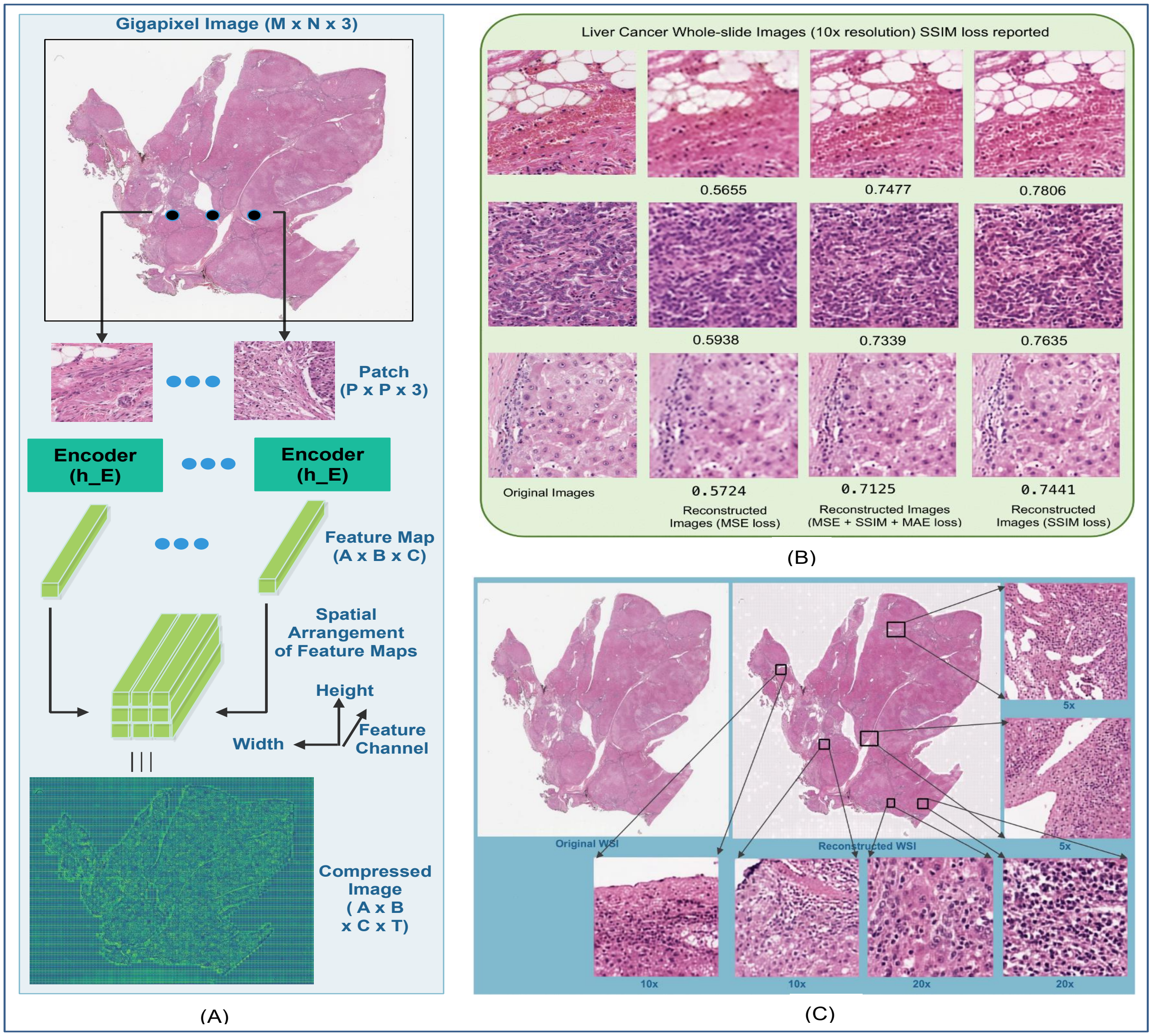

Figure 1: Gigapixel histology image compression. Top: a Gigapixel whole-slide histopathology image is divided into a set of small patches each of which are mapped to a low-dimensional feature maps using the autoencoder (the encoder h_E). Center: these embeddings are stored keeping the spatial arrangement of the original patches. Bottom: the resulting feature map as a compressed representation of the gigapixel image. M and N: size of the gigapixel image; P: patch size (256×256 in our study); This figure is referenced from [13]

Table 1: HistoCAE Architecture

	Layer	Layer types	Filter size	Activation	stride	Feature map dimension
Encoder	0	Input	-	-	-	256x256x3
	1	Convolution	3x3x16	Relu	1	256x256x16
	2	Batchnormalization	-	-	-	256x256x16
	3	Convolution	3x3x16	Relu	2	128x128x16
	4	Batchnormalization	-	-	-	128x128x16
	5	Convolution	3x3x32	Relu	1	128x128x32
	6	Batchnormalization	-	-	-	128x128x32
	7	Convolution	3x3x32	Relu	2	64x64x32
	8	Batchnormalization	-	-	-	64x64x32
	9	Convolution	3x3x64	Relu	1	64x64x64
	10	Batchnormalization	-	-	-	64x64x64
	11	Convolution	3x3x64	Relu	2	32x32x64
	12	Batchnormalization	-	-	-	32x32x64
	13	Convolution	3x3x64	Relu	1	32x32x64
	14	Batchnormalization	-	-	-	32x32x64
	15	Convolution	3x3x64	Relu	2	16x16x64
	16	Batchnormalization	-	-	-	16x16x64
Decoder	17	Convolution	3x3x64	Relu	1	16x16x64
	18	Batchnormalization	-	-	-	16x16x64
	19	Convolution	3x3x64	Relu	1	16x16x64
	20	Batchnormalization	-	-	-	16x16x64
	21	Upsampling	2x2	-	-	32x32x64
	22	Convolution	3x3x64	Relu	1	32x32x64
	23	Batchnormalization	-	-	-	32x32x64
	24	Convolution	3x3x64	Relu	1	32x32x64
	25	Batchnormalization	-	-	-	32x32x64
	26	Upsampling	2x2	-	-	64x64x64
	27	Convolution	3x3x32	Relu	1	64x64x32
	28	Batchnormalization	-	-	-	64x64x32
	29	Convolution	3x3x32	Relu	1	64x64x32
	30	Batchnormalization	-	-	-	64x64x32
	31	Upsampling	2x2	-	-	128x128x32
	32	Convolution	3x3x16	Relu	1	128x128x16
	33	Batchnormalization	-	-	-	128x128x16
	34	Convolution	3x3x16	Relu	1	128x128x16
	35	Batchnormalization	-	-	-	128x128x16
	36	Upsampling	2x2	-	-	256x256x16
	37	Convolution	3x3x3	Sigmoid	1	256x256x3
Classification		Input	-	-	-	16x16x64
	38	Convolution	3x3x128	Relu	2	8x8x128
	39	Batchnormalization	-	-	-	8x8x128
	40	Flatten	-	-	-	8192
	41	Dense	4096	Relu	-	4096
	42	Batchnormalization	-	-	-	4096
	43	Dropout (0.7)	-	-	-	4096
	44	Dense	4096	Relu	-	4096
	45	Batchnormalization	-	-	-	4096
	46	Dropout (0.7)	-	-	-	4096
	47	Dense	256	Relu	-	256
	48	Batchnormalization	-	-	-	256
	49	Dropout (0.7)	-	-	-	256
	50	Dense	2	Softmax	-	2

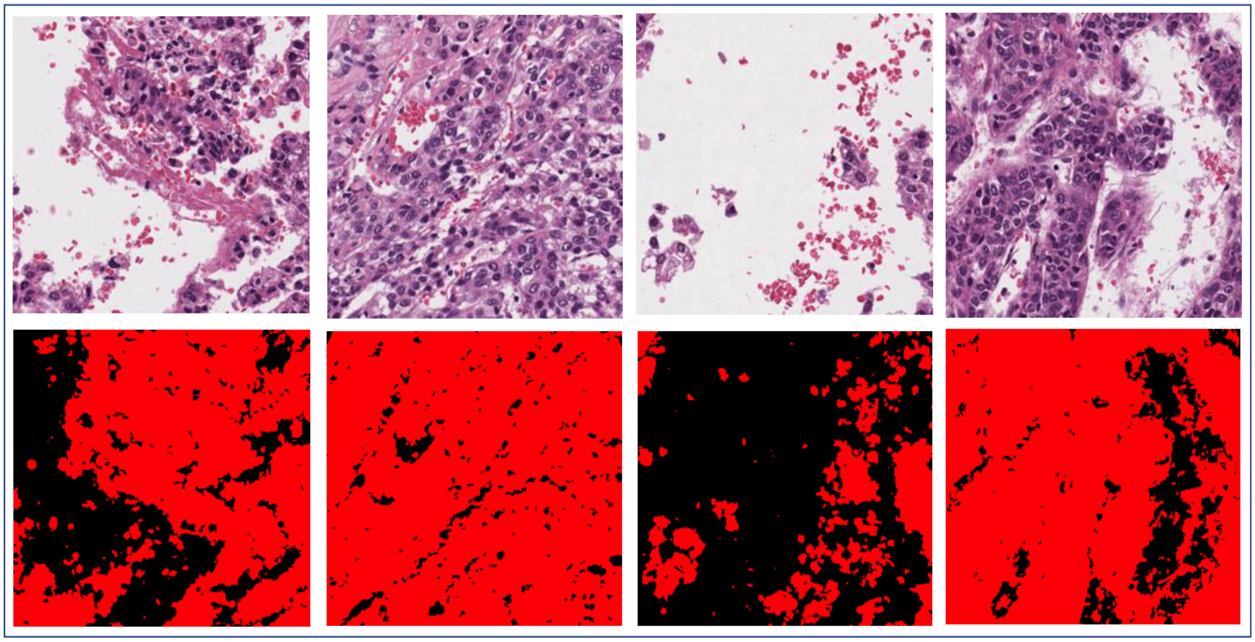

Figure 2: Representative patch wise viable tumor ground truth masks with presence of a large number of complex vasculatures. The top row presents the original tissue image patches and the bottom row presents the corresponding ground truth masks. Tumors and background are represented by red and black masks, respectively. Highly irregular vasculatures in the tumor masks are observed along with a large number of internal discontinuous spaces.

Table 2: Comparisons of the HistoCAE model performance across 5x, 10x and 20x, respectively.

Model Name	Test Accuracy	Precision		Recall		F1-Score		Proportion of correct patches	Dice Similarity
		Non-tumor	Tumor	Non-tumor	Tumor	Non-tumor	Tumor		
HistoCAE1 10x resolution (Our model)	0.95	0.96	0.93	0.95	0.95	0.96	0.94	0.94	0.87
HistoCAE1 5x resolution	0.94	0.93	0.94	0.99	0.87	0.96	0.92	0.94	0.63
HistoCAE1 20x resolution	0.89	0.87	0.95	0.98	0.75	0.92	0.84	0.89	0.47

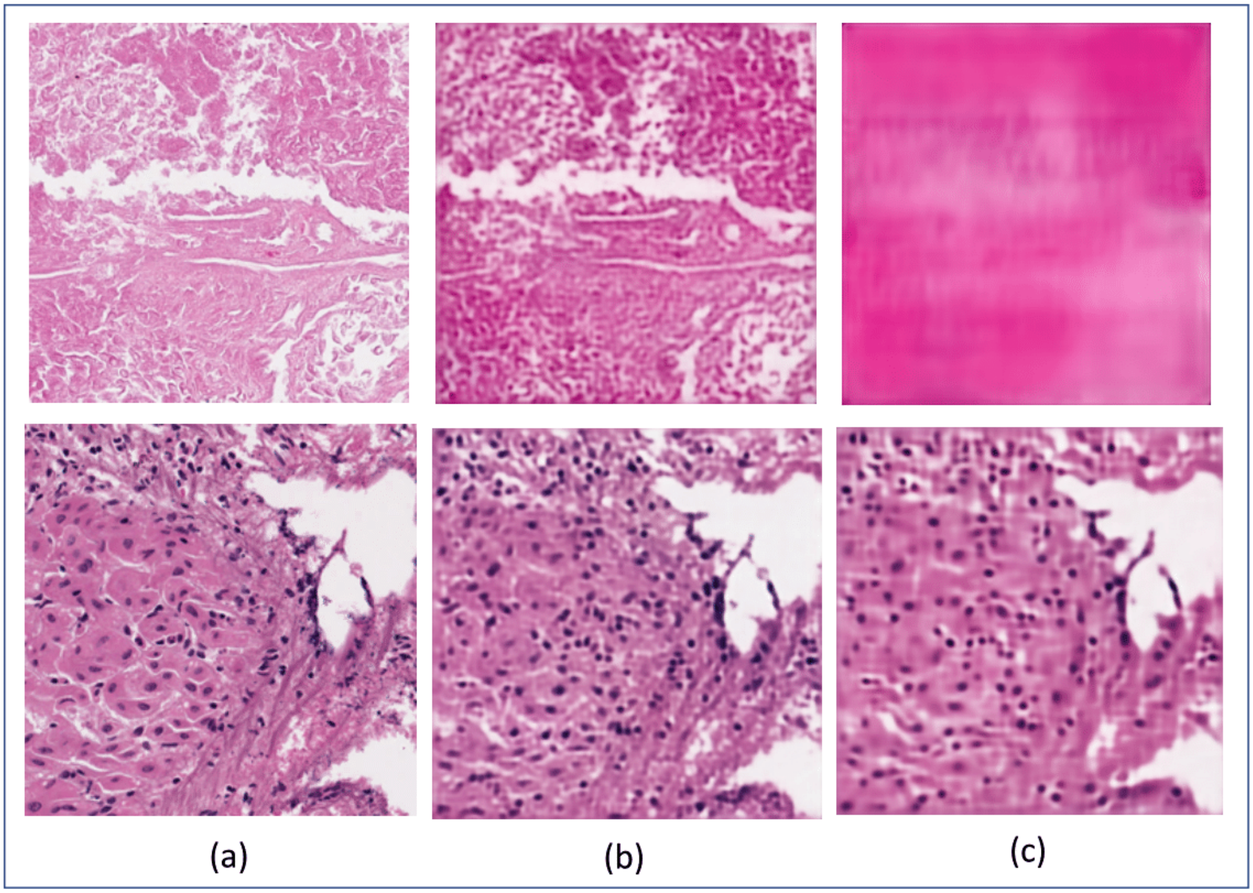

Figure 3: Comparisons of representative reconstruction results of (a) original images with (b) convolution (stride 2), and (c) max pooling.

Table 3: Performance comparison between HistoCAE at 10x and 20x magnification and MR-HistoCAE model at 20x magnification.

Model Name	Test Accuracy	Precision		Recall		F1-Score		Proportion of correct patches
		Non-tumor	Tumor	Non-tumor	Tumor	Non-tumor	Tumor	
HistoCAE1 (MSE + SSIM + MAE loss) 10x resolution	0.95	0.96	0.93	0.95	0.95	0.96	0.94	0.94
HistoCAE1 (MSE + SSIM + MAE loss) 20x resolution	0.89	0.87	0.95	0.98	0.75	0.92	0.84	0.89
MR-HistoCAE 20x resolution	0.94	0.94	0.92	0.96	0.90	0.95	0.91	0.94

Table 4: Performance comparison between the strided convolution and max pooling.

Model Name HistoCAE1	Test Accuracy	Precision		Recall		F1-Score		Proportion of correct patches	Dice Similarity
		Non-tumor	Tumor	Non-tumor	Tumor	Non-tumor	Tumor		
Strided-Conv Encoder Module	0.95	0.96	0.93	0.95	0.95	0.96	0.94	0.94	0.87
Max-Pooling Encoder Module	0.93	0.93	0.92	0.94	0.92	0.93	0.92	0.93	0.75

Table 5: Statistical analysis is shown by ANOVA and Kruskal-Wallis method. P-value is reported for each performance metric. Here we have compared all segmentation and classification methods together. We performed one-way ANOVA and Kruskal-Wallis for each metric grouped by all models together.

	Statistical Test	Test Accuracy	Precision	Recall	F1-Score	Dice Similarity
ANOVA (parametric method)	p-values	$<<0.001$	$<<0.001$	$<<0.001$	$<<0.001$	0.14
Kruskal-Wallis (non-parametric method)	p-values	$<<0.001$	$<<0.001$	$<<0.001$	$<<0.001$	$<<0.001$

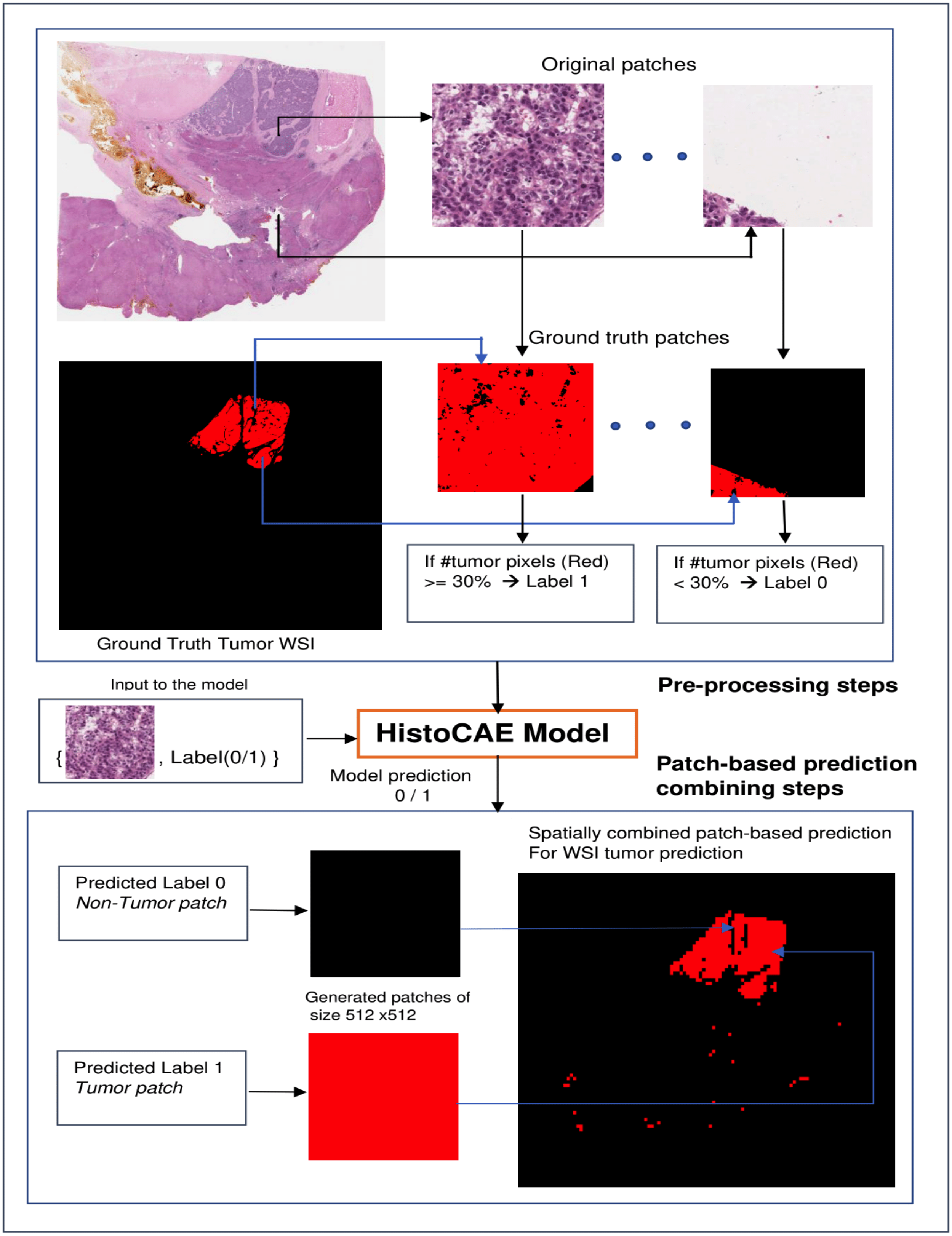

Figure 4: Workflow diagram representing the pre-processing steps, HistoCAE model prediction, followed by combining the patch-based prediction result to generate WSI tumor segmentation result.

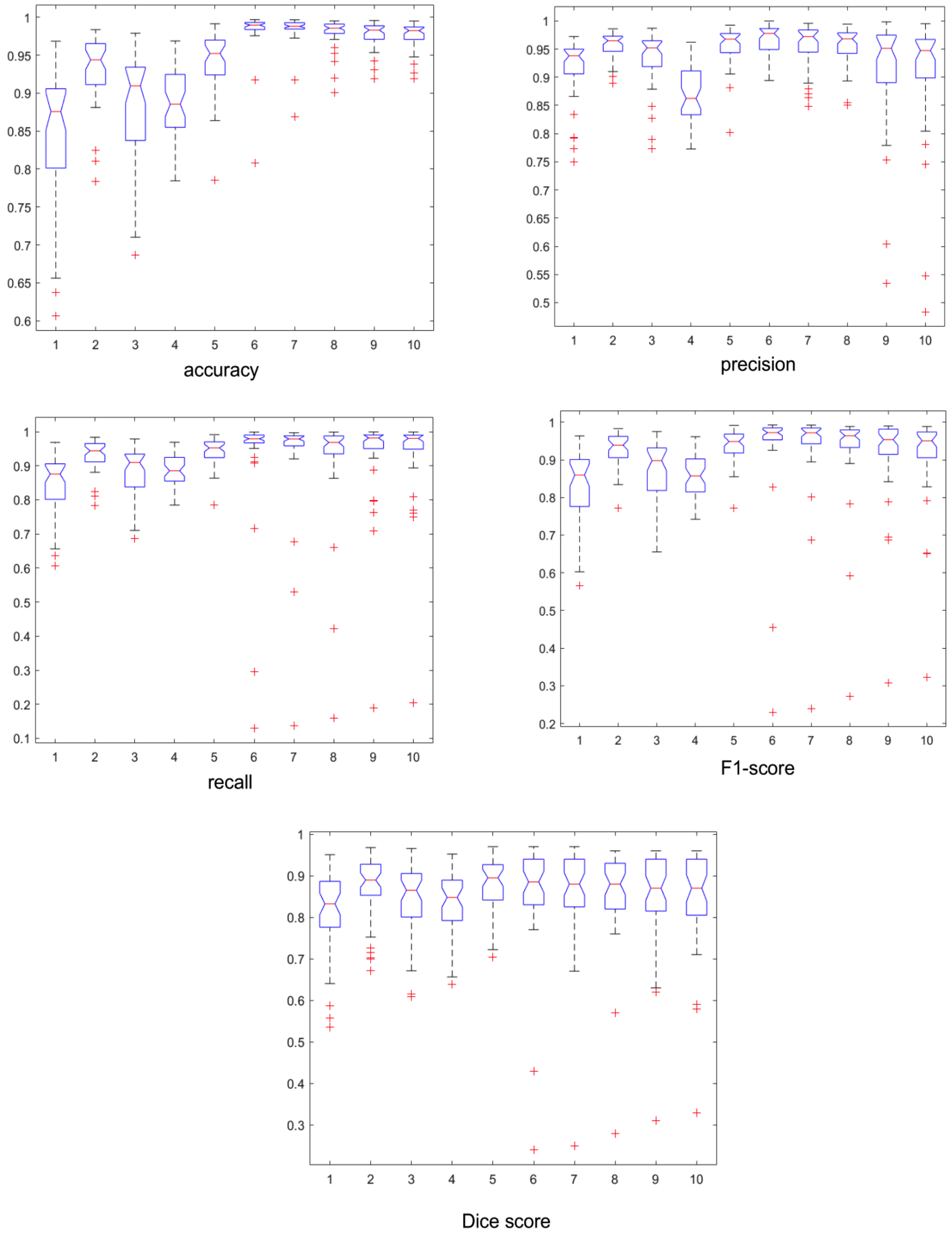

Figure 5: Box plot for each metric: test accuracy, precision, recall, F1-score, Dice similarity coefficient from DeepLabV3(1), MobileUNet(2), PSPNet(3), SegNet(4), RefineNet(5), DenseNet(6), Inception V3(7), ResNet101(8), Histo-CAE1(our model)(9) and HistoCAE2 with only MSE loss (10) respectively.
